# Ecotourism as a source of infection with *Schistosoma mansoni* in Minas Gerais, Brazil

**DOI:** 10.1186/s40794-016-0019-8

**Published:** 2016-02-24

**Authors:** Felipe Leão Gomes Murta, Cristiano Lara Massara, Joyce Favacho Cardoso Nogueira, Omar dos Santos Carvalho, Cristiane Lafetá Furtado de Mendonça, Viviane Aparecida Oliveira Pinheiro, Martin Johannes Enk

**Affiliations:** 1 Laboratory of Environmental and Health Education, Oswaldo Cruz Institute, Fiocruz, Brazil; 2 Laboratory of Helminthology and Medical, Malacology-René Rachou Research Center, Fiocruz, Minas Brazil; 3 Laboratory of Intestinal Parasites, Schistosomiasis and Malacology, Evandro Chagas Institute, Ananindeua, Brazil & Secretary of Health Surveillance, Ministry of Health, Rio de Janeiro, Brazil; 4 Superintendency of Health Surveillance, Municipal Secretary of Health, Ribeirão das Neves, Minas Gerais Brazil

**Keywords:** Schistosomiasis, Rural tourism, Outbreak

## Abstract

**Background:**

In recent years, a new pattern of schistosomiasis transmission has been described which is related to recreational activities associated with rural or ecological tourism and migratory flows and accompanying changes in social dynamics in Brazil. The objective of this report is to describe two schistosomiasis outbreaks that occurred during the practice of rural tourism in Minas Gerais, Brazil, and review this pattern of transmission within the wider context of schistosomiasis control.

**Findings:**

The first outbreak was characterized by its high infection rate, showing that 59 % of the exposed eco-tourists became positive for infection with *Schistosoma mansoni*. In addition, all three disease transmitting species of intermediate host snails were found in the area. In the second outbreak, all members of one tourist family were infected and reported contact with water in a well-known tourist area. The malacological survey in the region revealed an infection rate with *S. mansoni* of 8.3 % among the collected snails.

**Conclusions:**

Infection of urban dwellers that report contact with contaminated water associated with ecotourism represents a new pattern of disease transmission and dissemination. The infection with the disease at these occasions finds its expression in outbreaks of acute schistosomiasis among internal tourists to rural areas. Therefore, epidemiological surveillance in endemic areas should be aware of this schistosomiasis transmission pattern, and a multidisciplinary approach, most of all sanitation and health education measures, is required in order increase the efficiency of control strategies.

## Findings

Outbreaks of urban schistosomiasis in endemic regions have been reported in recent years [1–3], predominantly characterized by clinical manifestations of acute phase of the disease, characterized by nonspecific symptoms and in many cases confounded with other infectious diseases. [4] In these specific cases, schistosomiasis transmission is often related to recreational activities associated with the “boom” of rural and ecological tourism and the migratory flows, from people who live in the big urban centers [4–6]. Recently, a significant increase in the intensity of urban-rural migration has been observed [7], which goes along with a change in the social perception of rural areas, such as environmental conservation, eco- and rural tourism and the maintenance of cultural heritage. Thus, people living in big urban centers are traveling to nearby villages in these rural areas where natural water resources are a great attraction for leisure purposes. In this paper, two outbreaks of acute schistosomiasis among residents of an urban center in the state of Minas Gerais, Brazil are described.

### Outbreak 1: Schistosomiasis caused by intra city tourism

The Epidemiological Surveillance Service of the Ribeirão das Neves municipality was notified about the case of a female child, aged 12, hospitalized with initial suspicion of dengue hemorrhagic fever and later clinically diagnosed as a case of acute schistosomiasis based on the symptoms diarrhea, fever and headache, and 70 % eosinophilia in a white blood cell count. The clinical diagnosis of infection with *S. mansoni* was confirmed by the detection of eggs in a qualitative stool test. A follow-up epidemiological case-finding survey revealed that 34 people, including family members and cohabitants, aged between 4 and 49 years, had water contact in the rural area of the city during a tour in the carnival season. Of these 34 individuals investigated, 16 described the emergence of intense itching after water contact suggesting symptoms assocated with penetration of cercariae. After a period of 3 weeks, symptoms characterized by abdominal pain, nausea, vomiting and diarrhea appeared. Stool examination of all members of the group confirmed 20/34 positive samples for infection with *S. mansoni*, indicating an attack rate of 59 %. The positive patients were treated with praziquantel in a single dose 60 mg/kg for adults and 50 mg/kg for children and four months after treatment all patients showed negative results for *S. mansoni* eggs in their stools. In addition, a malacological survey was carried out at the body of water and surrounding areas where the exposure occurred, resulting in the capture of 348 snails of the genus *Biomphalaria*, the intermediate host of the parasite. It is also important to note that this body of water has untreated human waste as part of its collection system. Identification by morphological and molecular analysis [8, 9], and examination for elimination of cercariae under artificial light and crushing, revealed the presence all three disease transmitting species of *Biomphalaria*, namely *B. glabrata, B. tenagophila and B. straminea,* though no infection of the snails with *S. mansoni* larvae.

### Outbreak 2: Schistosomiasis caused by inter-city tourism

The Epidemiological Surveillance Service of the Ribeirão das Neves municipality again was informed about the occurrence of another six cases of acute schistosomiasis among members of a single family with ages ranging between 16 and 80 years. The family members showed symptoms similar to those in the first outbreak and were diagnosed, treated and followed up as described above. They reported contact with a water in small stream that is often used for recreational activities in the city of Santana do Riacho, an important ecotourism resort in the Serra do Cipó National Park in Minas Gerais, Brazil. In response to this cluster, 24 snails from the area of exposure were collected and identified as *B. glabrata*. Exposition to artificial light for detection of infection with *S. mansoni* confirmed that two (8.3 %) of the snails were eliminating cercariae.

### Discussion and implications

It is noteworthy that infection dynamics of these two outbreaks shows similarity, involving urban family households that travel for leisure purposes to ecotourism areas with natural water source contact exposure. In the first outbreak, autochthonous infection, by way infection within the municipality, was observed in a water collection system which is contaminated with untreated human waste and showed the presence of vector competent water snails. The contamination of the aquatic environment with human feces in combination with the presence of the intermediate host snails provides the ideal environment for disease transmission and consequently infection of visitors to this area who may have leisure and recreational water contact.

The second outbreak calls attention to the encounter of infected snails in a well-known tourist area. This region receives thousands of visitors (both local and outside) involving ecotourism associated with natural water activities. Schistosomiasis cases and infected snails in the region have previously been reported [6, 10], and our findings corroborate and document continued active transmission of schistosomiasis in this area and risk to tourists. This region is part of a mountainous complex do Espinhaço and is considered a natural reserve of extreme hydrographic importance to the State of Minas Gerais. The government in partnership with the private sector has initiated specific programs to stimulate international tourism growth to the region [11]. Although there is general knowledge that this area is endemic for schistosomiasis, little is done to sensitize the local community to the problem and to report on the risks of disease transmission to tourists [6]. This situation reveals the need for a coordinated planning effort in the development of the tourism sector, including mainly reorganization and restructuring of sanitation measures, such as waste disposal and sewage treatment, as well as the implementation of integrated health educational programs for the local community and visiting tourists regardless of the country of origin.

It is well-known that water contact and consequently infection exposure differs between residents of endemic areas and travelers and tourists to these areas [REFERENCE NEEDED]. It is also important to note that the immune response to the parasite in tourists or travelers is different from that of residents in endemic areas, causing clinically more severe symptoms among tourists when first exposed to the parasite, and possibly more outbreaks of acute schistosomiasis being identified among large groups of travelers [12]. Considering global climate change, Brazil currently lives a water crisis that affects the major urban centers as well as tourist areas as described. People in their search for water resources, both for consumption and for recreation, end up exposing themselves to unsafe water, as these sites do not always provide adequate sanitation of sources, and put themselves at the risks for infection not only with schistosomiasis but also with other water borne diseases, favoring their further spread and hampering already insufficient control efforts. In the context of the recent publication “Time to set the agenda for schistosomiasis elimination” by Rollinson and colleagues [13], social and environmental determinants of schistosomiasis have to be considered for effective control strategies, including a transversal dialogue with populations at risk, informing and educating about the factors and environment linked to schistosomiasis transmission and to the parasite’s life cycle, without stigmatization or imposition. The implementation of such strategies remains a great challenge in controlling the disease in Brazil.

## References

[1] Barbosa CS, Leal-Neto OB, Gomes ECS, et al. The endemisation of schistosomiasis in Porto de Galinhas, Pernambuco, Brazil, 10 years after the first epidemic outbreak. Mem Inst Oswaldo Cruz. 2011; doi:S0074-02762011000700014. 10.1590/s0074-02762011000700014 22124561

[2] Blanton RE, Barbosa LM, Reis E, et al. The Relative Contribution of Immigration or Local Increase for Persistence of Urban Schistosomiasis in Salvador, Bahia, Brazil. PLOS Neglected Tropical Diseases. 2015; doi:9:e0003521. 10.1371/journal.pntd.0003521 PMC4361398 25775457

[3] Ximenes RADA, Southgate B, Smith PG, Guimarães Neto L. Migration and urban schistosomiasis. The case of São Lourenço da Mata, Northeast of Brazil. Rev Inst Med Trop São Paulo. 2000; doi:10.1590/S0036-46652000000400006. 10.1590/s0036-46652000000400006 10968884

[4] Enk MJ, Amorim A, Schall VT. Acute Schistosomiasis Outbreak in the Metropolitan Area of Belo Horizonte, Minas Gerais: Alert about the Risk of Unnoticed Transmission Increased by Growing Rural Tourism. Mem Inst Oswaldo Cruz. 2003; doi:10.1590/S0074-02762003000600006. 10.1590/s0074-02762003000600006 14595449

[5] Enk MJ, Caldeira RL, Carvalho OS, Schall VT Rural tourism as risk factor for the transmission of schistosomiasis in Minas Gerais, Brazil. Mem Inst Oswaldo Cruz. 2004;doi:10.1590/S0074-02762004000900019. 10.1590/s0074-02762004000900019 15486645

[6] Massara CL, Amaral GL, Caldeira RL, et al. Schistosomiasis in ecotourism area of Minas Gerais, Brazil. Cad Saude Publica. 2008; doi:10.1590/S0102-311X2008000700025. 10.1590/s0102-311x2008000700025 18670694

[7] Skeldon R (1997). Migration and Development: A Global Perspective.

[8] Paraense WL (1975). Current status of systematic Brazilian planorbids. Arq Mus Nac RJ.

[9] Vidigal TH, Spatz L, Nunes DN, et al. *Biomphalaria* spp: identification of the intermediate snail hosts of *Schistosoma mansoni* by polymerase chain reaction amplification and restriction enzyme digestion of the ribosomal RNA gene intergenic spacer. Exp Parasitol.1998; doi:10.1006/expr.1998.4286. 10.1006/expr.1998.4286 9635441

[10] Massara CL, Dos Santos Carvalho O, Caldeira RL, et al. First report on the presence of *Biomphalaria straminea* in the municipality of Jaboticatubas, State of Minas Gerais, Brazil. Mem Inst Oswaldo Cruz. 2002; doi:10.1590/S0074-02762002000900007. 10.1590/s0074-02762002000900007 12426590

[11] Ruschmann DM. Ecological tourism in Brazil. Tour Manag.1992; doi:10.1016/0261-5177(92)90048-C.

[12] Clerinx J, Van Gompel A. Schistosomiasis in travelers and migrants. Travel Med Infect Dis. 2011; doi:10.1016/j.tmaid.2010.11.002. 10.1016/j.tmaid.2010.11.002 21216199

[13] Rollinson D, Knopp S, Levitz S, et al. Time to set the agenda for schistosomiasis elimination. Acta Tropica. 2012; doi:10.1016/j.actatropica.2012.04.013. 10.1016/j.actatropica.2012.04.013 22580511

